# Abnormal resting-state effective connectivity of triple network predicts smoking motivations among males

**DOI:** 10.3389/fpsyt.2025.1622162

**Published:** 2025-08-14

**Authors:** Mengzhe Zhang, Jieping Sun, Qiuying Tao, Jinghan Dang, Weijian Wang, Shaoqiang Han, Yarui Wei, Jingliang Cheng, Yong Zhang

**Affiliations:** Department of Magnetic Resonance Imaging, The First Affiliated Hospital of Zhengzhou University, Zhengzhou, China

**Keywords:** tobacco use disorder, triple network, smoking motivations, dynamic causal modeling, effective connectivity

## Abstract

**Background:**

The causal or direct connectivity alterations of triple network including salience network (SN), central executive network (CEN), and default mode network (DMN) in tobacco use disorder (TUD) and the neurobiological features associated with smoking motivation are still unclear, which hampered the development of a targeted intervention for TUD.

**Method:**

We recruited 93 male smokers and 55 male non-smokers and obtained their resting-state functional MRI (rs-fMRI) and smoking-related clinical scales. We applied dynamic causal modeling (DCM) to rs-fMRI to characterize changes of effective connectivity (EC) among seven major hubs from triple networks in TUD. Leave-one-out (LOO) cross-validation was used to investigate whether the altered EC could predict the smoking motivations (evaluated by Russell Reason for Smoking Questionnaire).

**Results:**

Compared with the control group, the TUD group displayed inhibitory extrinsic effective connectivity within SN. The abnormal ECs between networks were mainly characterized by uncoordinated switching between DMN and ECN activities in TUD individuals, with insula acting as a causal hub in this process. Moreover, increased EC from the right dorsolateral prefrontal cortex (R-DLPFC) and medial prefrontal cortex (MPFC) could predict the smoking motivations related to physical dependence.

**Conclusions:**

This study revealed aberrant causal connectivity in triple network and clarified the potential neural mechanism of smoking behavior driven by physical dependence. These findings suggested that a network-derived indicator could be a potential bio-marker of TUD and help to identify the heterogeneity in the motivation of smoking behavior.

## Introduction

Tobacco use disorder (TUD) is a chronic and recurring psychiatric disorder marked by diminished inhibitory control and a compulsive persistent smoking behavior (1, 2). The motivations for smoking are diverse and have significant individual heterogeneity, mainly including psychological and physical dependence, which related to different stages of addiction. The efficacy of current treatments for TUD remains unsatisfactory—for instance, varenicline, acting as a partial agonist of the α4β2 nicotinic acetylcholine receptor, is applied to TUD treatment but achieves only a 30% success rate in helping individuals quit (3), which emphasizes the limited understanding in the etiology and pathophysiology of TUD. Previous research have reported that executive and salience network presented a blunted response during social–emotional tasks and demonstrated increased activation during exposure to drug-related cues in substance addicts (4). However, the exact mechanism of TUD induced by diverse motivations is still unknown.

TUD is a multifaceted addictive disorder, distinguished by abnormalities in both the function and structure of specific brain areas (5). Previous research found that TUD individuals displayed decreased local functional connectivity (measured by regional homogeneity (ReHo)) in the inferior frontal cortex and higher local functional connections in the superior parietal lobe compared with controls (6). In addition to these localized or single connections, substantial interactions occur both within and across the primary core neurocognitive networks (7–9). Menon’s study clarified a triple network model for neuropsychiatric diseases and concentrated on the manner in which disruptions in widespread brain areas functioned within large-scale network systems, including default mode network (DMN), salience network (SN), and central executive network (CEN) (10). The posterior cingulate cortex (PCC) and the medial prefrontal cortex (MPFC) are the key nodes of DMN and participated in self-referential process and coordinating brain endogenous activity in resting-state (11, 12). CEN is involved in several high-level brain tasks, especially cognitive control and decision-making process (13, 14). SN, including anterior cingulate cortex (ACC) and anterior insula, dynamically distributes attentional and cognitive resources between the CEN and DMN to facilitate the transition between varying physical states (10, 15). This kind of systematic connection within or between networks has proven to be an anomaly in other substance use disorders, such as with cannabis. Ma’s study demonstrated that the cannabis use group exhibited aberrant effective connectivity within SN and between the DMN and SN regions (insula and PCC) relative to the control group (16). A previous study has shown a dysfunction in the MPFC–PCC–inferior parietal lobe loop within DMN among TUD individuals (17). However, the malfunction in one key network may affect the functioning of other interconnected networks (18). Thus, a comprehensive in-depth understanding of TUD from the perspective of integrated network interactions is needed. Moreover, the CEN and SN exhibited a blunted response during social–emotional tasks and showed increased engagement when exposed to drug cues among individuals with substance addiction (4). Heavy smokers, but not light smokers, showed decreased functional connectivity between SN and DMN and showed higher functional connectivity between CEN/DMN and SN after smoking replenishment (19). So, we hypothesized that the disconnection of triple network was related to the different levels of severity of TUD and induced by different motivations.

Previous studies investigated the abnormal intrinsic connectivity of TUD by using functional connectivity (FC) method. Shen’s study indicated that smokers showed a significantly decreased FC between bilateral Crus I and the brain areas involved in DMN, sensorimotor area and prefrontal cortex, compared with the controls (20). During the first day of quitting, smokers who could resist smoking showed significant FC between the left anterior insula (LAI) and the dorsolateral prefrontal cortex (DLPFC), while there was no such connectivity in relapse (21). However, while FC can quantify the statistical relationships between neurophysiological signals, it is unable to reveal the directed effective/causal effects that are behind these relationships (22). The available findings could not support *sequitur* about a directed or causal connection among large-scale networks implicated by smoking. In order to solve the abovementioned limitations and further reveal the brain information flows, some researchers proposed to apply the dynamic causal modeling (DCM) method to rs-fMRI, called spectral DCM (23). Spectral DCM could not only estimate DCM parameters more efficiently but also detect group differences sensitively by using parametric empirical Bayes (PEB) analysis, which considers both the mean and variance of effective connectivity (EC) estimation to infer group differences (24, 25). This approach was recently used to explore the dysregulated cross-network interactions among SN, CEN, and DMN among schizophrenia patients and showed a great hypothesis testing capability (18). For TUD individuals, Tang et al. conduct a preliminary study of EC in DMN using spectral DCM, but the authors only focused on four key nodes of DMN to conduct the fully connected DCM modeling while ignoring the dysregulated cross-network interactions among SN, CEN, and DMN (17). The integration of functional networks and directed information flows among large-scale networks of TUD needs to be further understood.

In this current study, we aimed to assess causal or direct connectivity alterations focused on DMN, CEN, and SN networks among TUD individuals by using spectral DCM method. Then, we explored the relationship between the altered ECs with group difference and smoking-related scales. Finally, we explored whether such altered ECs could predict smoking motivations in TUD individuals. Previous studies have identified that alterations in functional connectivity within specific brain networks (including DMN, CEN, and SN) underlie the neurobiological basis of TUD, and the DLPFC played a critical role in cue-induced craving (26). Based on the studies reviewed above, we hypothesized that (i) individuals with TUD would exhibit abnormalities within the SN or between the DMN and SN regions compared with the control group, and these anomalies could be associated with the severity of addiction and (ii) the ECs related to CEN regions would predict the smoking behaviors driven by physical dependence.

## Methods

### Subjects

A total of 148 participants were recruited in Henan Province, China, including 93 smokers and 55 non-smoking healthy controls. All of the participants were male adults, right-handed, and aged from 18 to 55. The smokers were defined as individuals who have smoked at least 10 cigarettes per day in the past 2 years and met the DSM-V criteria for TUD (27). For the healthy controls, we recruited subjects who did not smoke or smoked less than five cigarettes up to now (28). The exclusion criteria for all subjects were as follows: (i) with a clinical diagnosis of neuropsychiatric diseases, such as schizophrenia, depression, and epilepsy, (ii) currently using psychotropic drugs or simultaneously abusing other addictive substances or drugs, such as heroin and alcohol, (iii) with organic brain lesions, or (iv) subjects who have contraindications to magnetic resonance imaging, such as claustrophobia, and postoperative implantation of ferromagnetic devices. The experiment was approved by the Medical Ethics Committee of First Affiliated Hospital of Zhengzhou University, and informed consent forms were obtained from each subject (2019-KY-297).

### Smoking-related clinical data

Demographic data were collected by two experienced physicians. We collected clinical scales and information related to smoking including the FTND scale, Russell Reason for Smoking Questionnaire (RRSQ) scale, age of onset, duration, number of cigarettes consumed per day, and the pack-year (years of smoking × cigarettes smoked per day/20). We used the FTND scale to measure the severity of TUD and used the RRSQ scale to investigate the motivations for smoking. There was a total of six items in the FTND scale: items 1, 3, and 5 assess the urgency of restoring the nicotine levels to a given threshold after nighttime withdrawal and items 2, 4, and 6 reflect the persistence of nicotine levels keeping around the threshold while awake (29). There was a total of 24 items in the RRSQ scale, including eight subscales. The eight subscales were divided into two dimensions: (i) psychosocial dimension related to psychosocial factors of smoking (subscales I–III) and (ii) pharmacological dimension related to substance dependence and addiction (subscales IV–VIII) (30).

### Image acquisition

MRI data were obtained using a 3.0-T German Siemens Magnetom Skyra magnetic resonance imaging equipment with a 16-channel prototype quadrature birdcage head coil at First Affiliated Hospital of Zhengzhou University, Henan Province, China. All smokers were required to smoke a cigarette within 30–45 min before entering the scanner to exclude withdrawal symptoms. The participants were instructed to rest with their eyes closed, to keep awake, to not think of anything, and to keep their head motionless during scanning. Earplugs were used to protect the hearing of the subjects, and spongy pads were used to fix their head to minimize head movement. No external stimuli was exerted during image acquisition. The parameters were repetition time (TR)/echo time (TE) = 2,000/30 ms, flip angle = 80°, matrix size = 64 × 64, field of view = 220 mm × 220 mm, voxel size = 3.4 mm × 3.4 mm × 4 mm, slices = 36, and slice thickness = 4 mm, with a total of 180 volumes. All slices along the AC–PC line were acquired with a total scan time of 360 s.

### Image preprocessing

In this study, data processing was conducted by using the DPARSF (data processing assistant for resting-state fMRI) toolbox based on MATLAB platform. It mainly included the following steps: First, format conversion (DICOM to NIFTI), discarding the first five volumes, slice timing, and realignment (cutoff < 2.5° or 2.5 mm). The images were spatially normalized to the standard EPI template and re-sampled to 3 × 3 × 3 mm. Functional images were spatially smoothed with a Gaussian kernel of full-width at half-maximum of 6 mm. Finally, linear detrending and scrubbing further eliminated the influence of head motion and noise.

### Dynamic causal modeling

**Figure 1:**
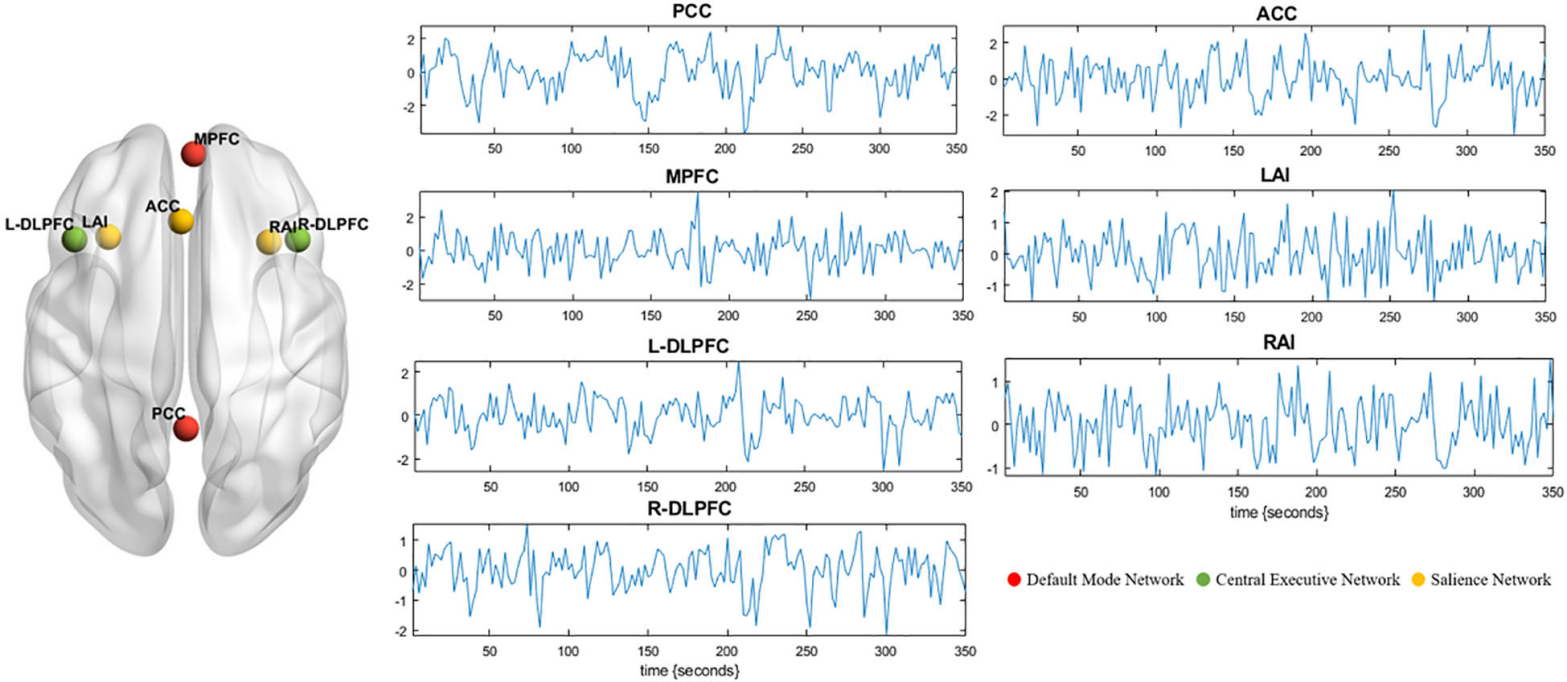
Seven ROIs are selected for DCM analysis. The left panel shows the spatial location of the ROIs. Red represents default mode network (MPFC and PCC). Green represents central executive network (L-DLPFC and R-DLPFC). Yellow represents salience network (ACC, LAI, and RAI). Blood-oxygen-level-dependent time series data of ROIs from one subject (middle and right panel). These time series were used to invert the spectral DCM with the (fully connected) architecture. DCM, dynamic causal modeling; ROI, region of interest; RAI, right anterior insula; LAI, left anterior insula; ACC, anterior cingulate cortex; PCC, posterior cingulate cortex; MPFC, medial prefrontal cortex; R-DLPFC, right dorsolateral prefrontal cortex; L-DLPFC, left dorsolateral prefrontal cortex.

Spectral DCM, as implemented in SPM12, was used for effective connectivity analysis. First, a general linear model was established in SPM with cosine basis function from 1/128 to 0.1 Hz as the interest effects and the movement parameters and with cerebrospinal fluid and white matter signals as nuisance regressors (31). Based on previous studies, a total of seven regions of interest (ROIs) were selected, including two DMN regions (medial prefrontal cortex [MPFC] and posterior cingulate cortex [PCC]), three SN regions (anterior cingulate cortex [ACC] and bilateral anterior insula [LAI/RAI]), and two CEN regions (bilateral dorsolateral prefrontal cortex [L-DLPFC/R-DLPFC]) (17, 32, 33). ROIs were defined as 6-mm-radius spheres centered at the spatial coordinates of Montreal Neurological Institute (MNI) reported in previous studies (for the MNI coordinate, see **Table 1**). Next, we extracted the time series signal of each ROI, and the center of the sphere was fixed. The signal and spatial position of ROIs are shown in **Figure 1**. For each ROI, the first principal component of the time series from all voxels included in the sphere was calculated (corrected for confounders). Without any external stimuli, a fully connected seven-node DCM model was built for each subject (including self-connection of each node, with a total of 49 connections). Then, the full DCM of each subject was inverted to obtain estimates of their free energy and the posterior probability (34). Finally, diagnostics of the model inversion were performed to assess the estimated parameters and the percentage variance explained by the model.

**Table 1:** MNI coordinates of seven ROIs.

Region	ROI abbreviation	X	Y	Z
Default mode network (17)				
Posterior cingulate cortex	PCC	0	-52	26
Medial prefrontal cortex	MPFC	3	54	-2
Central executive network (33)				
Left dorsolateral prefrontal cortex	L DLPFC	-43.4	20.9	38.1
Right dorsolateral prefrontal cortex	R DLPFC	43.4	20.9	38.1
Salience network (32)				
Anterior cingulate cortex	ACC	-2	28	28
Left anterior insular	LAI	-30	22	-6
Right anterior insular	RAI	32	20	-6

MNI, Montreal Neurological Institute; ROI, region of interest.

### Parametric empirical Bayes analyses

A group level analysis of each EC (a total of 49 tested connections) was performed using the parametric empirical Bayes (PEB) method (35). PEB is a between-subject hierarchical or empirical Bayesian model over parameters that models how individual connections relate to group or condition means. This hierarchical approach treats each connection as a random (between-subject) effect, which is modeled by adding a random Gaussian variation to subject-specific predictions based upon the group mean connectivity as well as between-subject effects (35). This parametric random effect modeling could use the full posterior density over the parameters from each subject’s DCM to inform the group-level result (36). This hierarchical modeling of random parametric effects could increase the sensitivity of the approach and renders it robust to outlier subjects with noisy data (35). To evaluate how the connectivity of TUD individuals differs from HCs, we then used Bayesian model reduction (BMR) to search over PEB models with different combinations of connections and group differences (37). BMR was used to iteratively prune connection parameters from the full PEB model, until model–evidence started to decrease. The parameters of the best 256 pruned models were then averaged, weighted by their evidence. In PEB, group-level analyses are conducted using Bayesian posterior inference, which does not need to contend with the multiple-comparison problem because of the lack of false positives (38). Bayesian posterior probability (Bayesian-PP) was used as an indicator of the confidence. The higher Bayesian-PP indicated the greater confidence. Here the results were considered reliable if Bayesian-PP >0.95 (16).

### Leave-one-out cross-validation

In order to investigate whether the altered EC could predict the motivations of smoking in each subject, we performed leave-one-out cross validation analysis to explore the relationship between RRSQ scores (including two dimensions) and ECs with group difference. A PEB model was fitted to all but one subject, and the RRSQ scores for the left-out subject were predicted, with age and years of education as covariates of non-interest (39). This was repeated with each subject left out, and the accuracy of the prediction was recorded. The Pearson correlation coefficient and normalized root mean squared error (NRMSE) were calculated between the actual and predicted scores to assess predictive performance (39).

### Statistical analyses

SPSS 22 software was used for data statistics. Clinical data was expressed as mean ± standard deviation. Two-sample *t*-tests were conducted to assess differences in age and years of education between the TUD group and the control group. Pearson’s correlations were performed to explore the relationships between altered ECs based on the PEB analysis and the severity of the disease, such as FTND score and pack-year.

## Results

### Demographic and clinical data

**Table 2** summarizes the demographics, clinical information related to smoking, nicotine dependence scores, and motivations for smoking. No significant differences are found in age and years of education between the TUD group and the control group.

**Table 2:** Demographic and smoking behavior.

Demographics	TUD (*n* = 93)	Healthy controls (*n* = 55)	*t* ^a^	*p*
Age (year)	34.1 ± 7.8	32.3 ± 7.4	1.434	0.154
Education (year)	15.1 ± 2.0	15.9 ± 3.1	-1.679	0.097
Age onset	19.1 ± 3.0	–	–	–
Smoking years	16.6 ± 7.2	–	–	–
Pack-year	18.1 ± 12.4	–	–	–
Cigarettes/day	21.0 ± 9.0	–	–	–
FTND_total_	4.2 ± 2.3	–	–	–
FTND_1,3,5_	2.3 ± 1.5	–	–	–
FTND_2,4,6_	1.9 ± 1.3	–	–	–
RRSQ_I–III_	8.4 ± 5.0	–	–	–
RRSQ_IV–VIII_	19.1 ± 7.7	–	–	–

Data represent mean ± standard deviation.

FTND, Fagerström Test for Nicotine Dependence; RRSQ, Russell Reason for Smoking Questionnaire; Pack-year, years of smoking × cigarettes smoked per day/20; TUD, tobacco use disorder.

a Two-sample *t*-test.

### Group comparison

**Figure 2:**
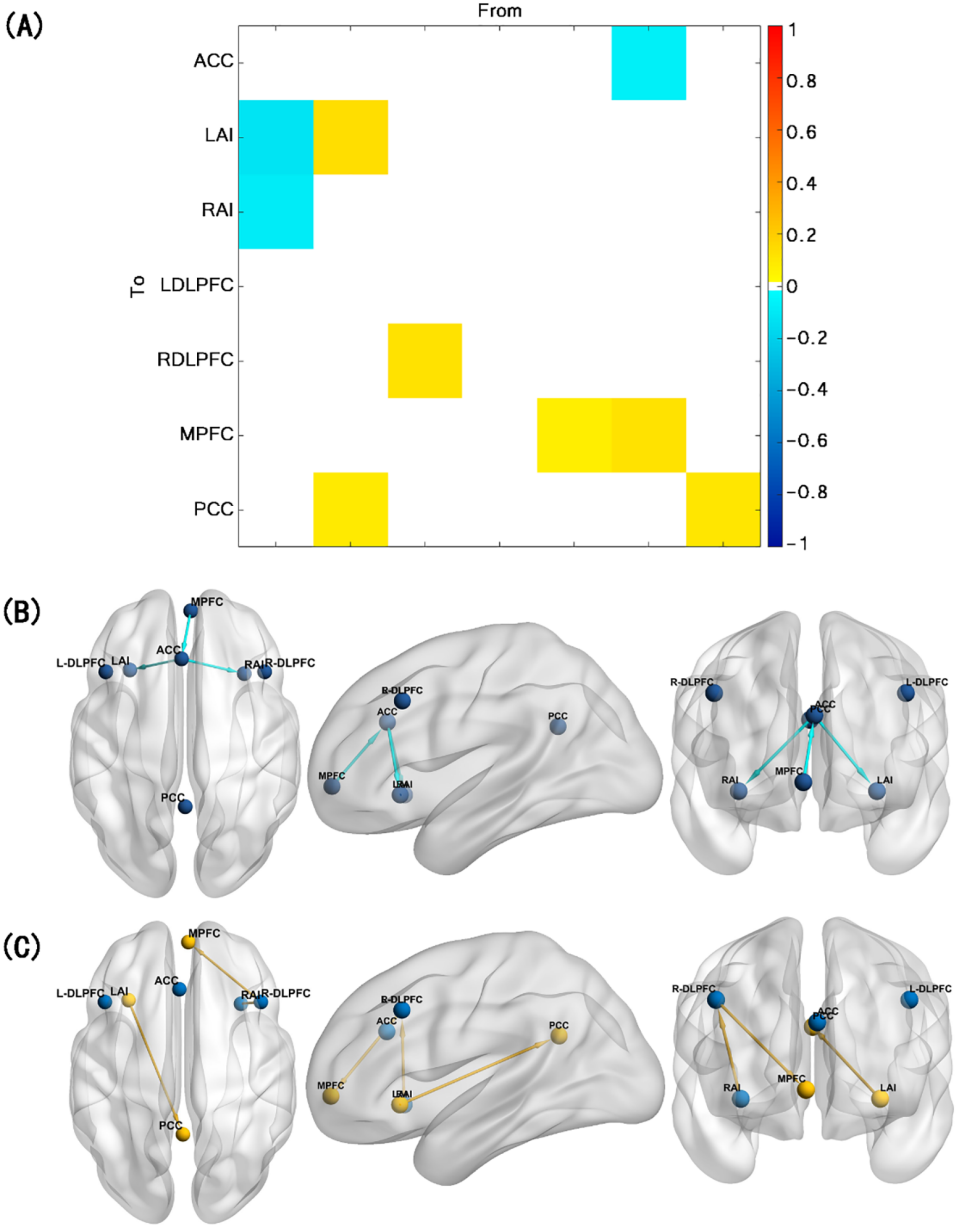
Results of the group comparison. **(A)** EC difference between TUD group and control group. **(B)** Decreased EC in TUD individuals compared with healthy controls (blue arrow). **(C)** Increased EC in TUD individuals compared with healthy controls (yellow arrow and yellow ball represent enhanced self-connection in this brain region). EC, effective connectivity; TUD, tobacco use disorder.

Compared with healthy controls, TUD individuals show increased ECs from RAI to R-DLPFC, R-DLPFC to MPFC, and LAI to PCC and decreased ECs from MPFC to ACC, ACC to LAI, and ACC to RAI (free energy, Bayesian-PP >0.95). Besides that, LAI, MPFC, and PCC show enhanced self-connections in TUD individuals (**Figure 2**). The group differences for each EC and corresponding Bayesian PP are shown in **Table 3**.

**Table 3:** EC difference between the TUD group and the control group.

Group	Connectivity	EC (HZ)	Bayesian-PP
TUD < HC	MPFC→ACC	-0.076	1.00
	ACC→LAI	-0.110	1.00
	ACC→RAI	-0.081	1.00
TUD > HC	RAI→R-DLPFC	0.116	1.00
	R-DLPFC→MPFC	0.077	1.00
	LAI→PCC	0.086	1.00
	LAI→LAI	0.134	1.00
	MPFC→MPFC	0.112	1.00
	PCC→PCC	0.099	1.00

EC, effective connectivity; PP, posterior probability; TUD, tobacco use disorder; HC, healthy control; RAI, right anterior insula; LAI, left anterior insula; ACC, anterior cingulate cortex; PCC, posterior cingulate cortex; MPFC, medial prefrontal cortex; R-DLPFC, right dorsolateral prefrontal cortex.

Arrow means EC from A to B.

### Leave-one-out cross-validation

**Figure 3:**
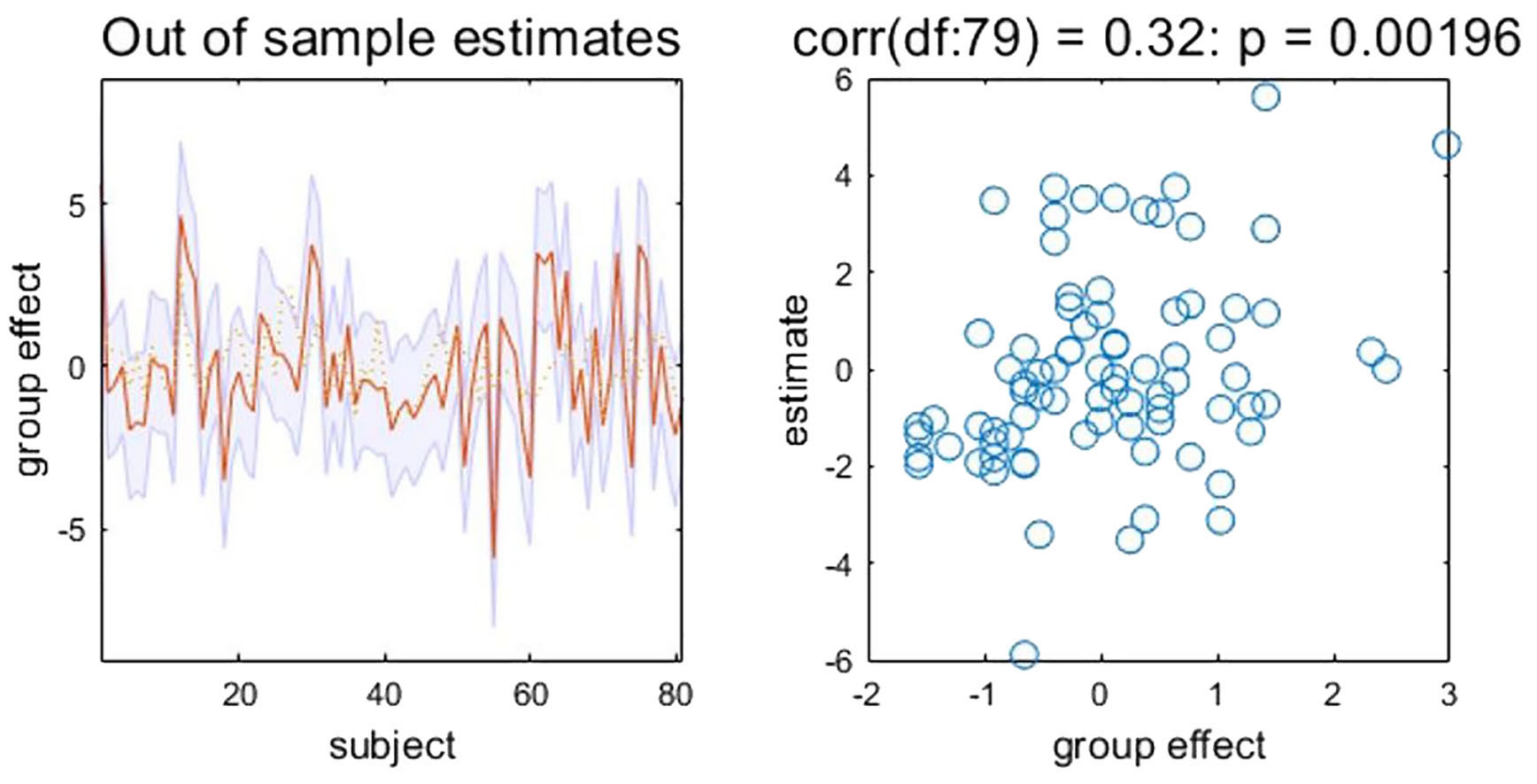
Leave-one-out cross-validation results. Left: Actual and (out‐of‐sample) predicted RRSQ_IV–VIII_ scores based on EC from R-DLPFC to MPFC (after mean correction and standardization). The yellow dashed line is the actual values. The red line is the predicted values. The shaded area represents 90% confidence interval. Right: Out-of-samples correlation of the actual RRSQ_IV–VIII_ scores against the predicted scores based on EC from R-DLPFC to MPFC for each left-out subject. EC, effective connectivity; MPFC, medial prefrontal cortex; R-DLPFC, right dorsolateral prefrontal cortex; RRSQ, Russell Reason for Smoking Questionnaire.

The RRSQ_IV–VIII_ scores predicted by EC from R-DLPFC to MPFC are significantly correlated with the actual scores in TUD individuals (*r* = 0.32, *p* = 0.002, NRMSE = 0.23, FDR-corrected) (see **Figure 3**). Therefore, the effect size estimated by DCM could predict the smoking motivations related to physical dependence.

### Correlation analyses

No significant correlations were found between altered ECs and severity of disease, including FTND score and pack-year (all *p*_corrected_ >0.05).

## Discussion

This study examined the anomalies in causal connections within triple network among TUD individuals by using spectral DCM. Compared with the control group, the TUD group displayed inhibitory extrinsic EC within SN and uncoordinated switching between DMN and CEN activities. Besides that, increased EC from R-DLPFC and MPFC could predict the smoking motivations related to physical dependence. These results help us further in realizing the neural mechanism of TUD and its interaction within and between triple networks.

### Self-connection changes in TUD

Firstly, we observed that the DMN and SN regions exhibited enhanced self-connection in TUD individuals compared with the control group. Given that the self-connection within the DCM is consistently inhibitory, the findings of increased self-inhibition in the DMN and SN regions suggest that these brain areas are more self-inhibited (17, 39). According to a previous study, it is probably due to the lower activation of these regions in chronic smokers (40). Interestingly, such self-inhibitory feature comes with an increased feedback connection between the inhibited nodes and other external nodes. Another potential biological explanation is that the heightened self-inhibition adjusts the balance between inhibitory and excitatory activities within each area, thereby preserving functional integration among specialized regions (41).

### EC aberrance within networks

Dysfunction of brain network (particularly SN) that mediates salience is crucial to the progression of TUD (42). SN is involved in integrating internal emotion (craving and withdrawal) and external stimuli (tobacco cues) to guide behaviors, which is associated with the motivation of smoking (43). Consistent with our findings, the self-inhibitory feature of the key nodes in SN (ACC and AI) implies inefficient internal/external information processing and cognitive resource allocation (10, 15). In terms of connections within network, we found decreased EC from ACC to bilateral AI in TUD individuals compared with healthy controls. The ACC and AI exhibit substantial topographical connectivity, forming a tightly integrated anatomical network, with highly specialized neurons (44). A previous study reported that young adult smokers exhibited decreased functional connectivity between ACC and right insula, and it showed an inverse relationship with the errors made during the Stroop color-word task, indicating compromised cognition controlling progress among smokers (45). The functional connectivity within SN exhibited varying characteristics across different smoking conditions, such as during nighttime abstinence (when deprived) and after smoking (when satiated) (46). After more than 12 h of abstinence, the connection between insula and ACC was highly increased in TUD individuals (19). On this basis, our findings further clarify the connection between ACC and AI is directional, and the EC from ACC to AI might be associated with craving and withdrawal of TUD.

### EC aberrance between networks

The dysfunction of SN could impact the other networks. The critical role of SN is to initiate network switching and allocate attentional and cognitive resources dynamically between the CEN and DMN to achieve the transformation of a different physical status (10, 15). Consistent with our hypothesis, we found disrupted EC between the DMN and SN regions in TUD individuals compared with healthy controls. One recent research proposed that the insula acted as a causal outflow hub to mediate dynamic switching between DMN and CEN activity (47). The activity in the insula being excessively coupled with the activity in DMN suggests that smokers indulge in more internal mental processes to deal with anxiety, stress, and other negative emotions (10). A previous study has demonstrated that during the initial phase of smoking abstinence, the interaction between the AI and DMN brain regions played a crucial role in redirecting attention to address the internal turmoil caused by nicotine deprivation. This shift in network dynamics biases cognitive processing toward the DMN while moving away from the CEN (48).

SN also acts on CEN; more specifically, TUD individuals showed that R-DLPFC has enhanced sensitivity to afferent coupling from the RAI compared with healthy controls. As discussed above, the dysfunction of SN results in compromised detection and mapping of salient external stimuli and internal events, leading to the abnormal activation of the CEN. This has substantial impacts on both cognitive processes and self-monitoring (10). The aberrance of CEN was thought to be associated with cognitive deficits in TUD individuals (49). Compared with neutral cues, TUD individuals showed increased bold activation in the right CEN when presented with smoking cues (50). Consistent with our findings, Wang’s research also demonstrated that smokers exhibited increased intrinsic connectivity between AI and CEN compared with non-smokers (47). A recent study has reported that smokers would show lower coupling between SN and DMN while demonstrating greater connections between SN and CEN when tobacco administration facilitated externally directed attention (48). Therefore, our results suggested that enhanced EC from SN (RAI) to CEN (R-DLPFC) in TUD individuals might be related to external stimulus-driven cognitive information processing, and more recruitment of CEN was needed in the face of external stimuli (especially smoking-related stimuli) as the severity of dependence increases (10, 51).

### EC predicted smoking motivations

Interestingly, the association between interactions of triple network and motivations for driving smoking behaviors was observed in TUD group. Our findings showed that EC from R-DLPFC to MPFC could predict RRSQ_IV–VIII_ scores in TUD individuals, which suggested that the adaptive modifications in this circuit could potentially serve as the neural biomarker underlying smoking behavior driven by physical dependence. The RRSQ subscales could assess an individual’s motivations for smoking, including psychological and physical dependence, which have been utilized to explore the interacting pathways among depressive symptom, stress, and smoking (30). The conventional understanding of addiction posits that tobacco use is largely governed by the level of nicotine in the body and directly related to the pharmacological effects of nicotine (52, 53), but a previous study has proposed the notion of social smokers who smoke exclusively in social settings and in the presence of other smokers, which may associate with their psychosocial image (54–56). Therefore, we hypothesized that multiple causes of smoking may be mediated by different neural circuits. The dual-system theory holds that the failure of self-control is caused by the conflict between automatic and deliberative modes of behavioral control (57). The DLPFC is responsible for governing and regulating action patterns as well as decision-making processes, while the MPFC plays a role in modulating limbic system activation and emotional processing (58, 59). The imbalance between the two systems is considered to play a role in increasing susceptibility to reinforce or relapse in addiction (57). Our current results lend support to the hypothesis that the abnormal communication of DLPFC and MPFC underlies the mediation of smoking behaviors associated with substance dependence and addiction rather than psychosocial factors.

### Significance

Dysfunctional connectivity patterns of DLPFC and MPFC may identify subgroups of smokers with distinct motivational drivers. A previous study has demonstrated that a transcranial magnetic stimulation (TMS) strategy targeting both the MPFC and DLPFC could significantly increase the 1-month abstinence rates and reduce cigarette consumption compared with sham treatment (59). Our findings provide a potential target to develop effective treatments for TUD individuals who smoke driven by physical dependence. Moreover, our study indicated the dysfunctional activity within SN uncoordinated switching between DMN and CEN activities in TUD, providing a rationale for individualized attentional bias training for SN dysregulation to address network-specific deficits. While large-scale implementation requires further validation, our study identifies a tractable target for pilot trials.

### Limitations

There are still some limitations in our research. First, despite uncovering the anomalies in causal connections within triple networks among TUD individuals, the current cross-sectional study is insufficient to explain how EC evolves over time and its relationship with clinical features over time. Second, gender differences of brain functional abnormalities are still unclear because only male subjects were recruited. A previous study has shown that male smokers are more sensitive to the reinforcing effects of tobacco than female smokers (60). Multicenter datasets and female subjects are needed to further verify these findings. Third, our current study did not stratify the participants based on the severity of disease. Future studies should focus on the abnormal communication patterns within/between the triple brain networks across individuals with TUD of different severity levels. Fourth, based on the current algorithm, the large number of connectivity parameters could inflate model complexity, leading to potential problems with overfitting. We only selected the key nodes of DMN, SN, and CEN as ROIs rather than all regions of networks. Future studies could constrain the number of external coupling parameters by using plausible priors, allowing for a more comprehensive evaluation of ECs in triple networks among TUD individuals (61, 62). Finally, evidence showed that reliability and similarity of functional connectivity estimates can be greatly improved by increasing the scan lengths from 5 min up to 13 min, but our study collected 6-min resting-state scanning (63).

## Conclusion

Overall, this study revealed aberrant causal connectivity in a large-scale brain organization focused on triple networks and emphasized their distinct roles of cognition and internal mental processes in TUD individuals. Our findings indicated the dysfunctional activity within SN uncoordinated switching between DMN and CEN activities in TUD and also clarified the pivotal role of AI in this process. Moreover, the abnormal communication of DLPFC and MPFC might be the basis of mediating smoking behavior driven by physical dependence rather than psychosocial factors. Network-derived indicators have the potential to serve as biomarkers for TUD and assist in elucidating the diverse motivations behind smoking behavior.

## Data Availability

The original contributions presented in the study are included in the article/supplementary material, further inquiries can be directed to the corresponding author/s.
